# Heterogeneity in Host Risk Factors for Incident Melanoma and Non-Melanoma Skin Cancer in a Cohort of US Women

**DOI:** 10.2188/jea.JE20100145

**Published:** 2011-05-05

**Authors:** Abrar A. Qureshi, Mingfeng Zhang, Jiali Han

**Affiliations:** 1 Channing Laboratory, Department of Medicine, Brigham and Women’s Hospital and Harvard Medical School, Boston, MA, USA; 2 Department of Dermatology, Brigham and Women’s Hospital and Harvard Medical School, Boston, MA, USA; 3 Department of Epidemiology and Biostatistics, Cancer Center, Nanjing Medical University, Nanjing, China; 4 Department of Epidemiology, Program in Molecular and Genetic Epidemiology, Harvard School of Public Health, Boston, MA, USA

**Keywords:** melanoma, squamous cell carcinoma, basal cell carcinoma, phenotype

## Abstract

**Background:**

Melanoma, squamous cell carcinoma (SCC), and basal cell carcinoma (BCC) are 3 types of skin cancer that have distinct biologic characteristics and prognoses. We evaluated phenotypic differences in the risk of these cancers in US women.

**Methods:**

We conducted a prospective study of 113 139 female nurses from 1984 to 2002. Over the 18 years of follow-up, there were 375 cases of melanoma, 495 cases of SCC, and 9423 cases of BCC.

**Results:**

Women with melanoma were more likely to have a family history of melanoma (melanoma: RR 1.94, 95% confidence interval [CI] 1.36–2.76; SCC: RR 0.82, 95% CI 0.58–1.37; BCC: RR 1.49, 95% CI 1.38–1.62) and 6 or more moles on the left arm (melanoma: RR 3.66, 95% CI 2.15–6.24; SCC: RR 1.53, 95% CI 0.83–2.79; BCC: RR 1.48, 95% CI 1.28–1.72). Polytomous logistic regression analysis showed that age at diagnosis (*P* < 0.0001), family history of melanoma (*P* = 0.016), and number of moles on the left arm (*P* = 0.007) were significantly different across the 3 cancers.

**Conclusions:**

This prospective observational study demonstrated that known phenotypic factors for skin cancer have a differential impact on the risk of melanoma, SCC, and BCC.

## INTRODUCTION

Skin cancer incidence is increasing in the Unites States, and better methods to identify at-risk individuals will support improved prevention strategies, especially for melanoma.^1^ Sun exposure is a known environmental cause of skin cancer.^2^ Established host risk factors for skin cancer include family history of skin cancer, fair skin color, inability to tan, susceptibility to burn, and light hair color.^3^^–^^5^ It has been suggested that there are phenotypic variables specific to melanoma, squamous cell carcinoma (SCC), and basal cell carcinoma (BCC).^5^ However, previous research compared findings across studies separately for melanoma, SCC, and BCC, which is problematic due to variations among populations and differences in study design. Therefore, it is worthwhile to conduct a study to evaluate known host risk factors across different histological types of skin cancer within the same population.

Tanning and burning are 2 distinct traits that dictate sun sensitivity and risk of skin cancer. However, they are often grouped in the assessment of Fitzpatrick skin type, a widely used method to determine skin cancer risk that uses a 6-point scale based on an individual’s past burning and tanning response to sun exposure.^6^ Recent findings on the differential pathways underlying burning and tanning responses has prompted researchers to ask whether sensitivity to burn and ability to tan are associated with differential risks for non-melanoma skin cancer and melanoma.^7^^,^^8^ Little has been reported on the impact of burning and the tanning response to sun exposure on the risks of melanoma, SCC, and BCC.

Hair color is highly correlated with baseline skin pigmentation, the tanning response, freckling, and susceptibility to burn.^9^^,^^10^ Two types of melanin pigment, eumelanin and pheomelanin, are recognized as critical determinants of individual photosensitivity (in addition to baseline skin and hair color) and contribute to tanning ability.^11^ Light hair color has been associated with increased risk of melanoma, SCC, and BCC.^5^

Acquired melanocytic nevi present in sun-exposed areas of the body, such as the extensor aspects of the upper extremities, have been associated with greater time spent in the sun during childhood and a higher risk of melanoma.^12^ The risk of melanoma in individuals with more than 100 nevi on their entire body has been shown to be 12 times that of individuals with fewer than 10.^13^ Similarly, multiple atypical nevi, multiple large nevi, and high nevus counts in sun-exposed or sun-protected areas have been associated with a high risk of melanoma. However, the risk of SCC and BCC associated with nevus counts has not been extensively studied.

Family history of melanoma is associated with a high risk of melanoma, independent of other phenotypic characteristics. Those with multiple primary melanomas tend to have a higher positive family history rate.^14^ There are few data on the risk of SCC and BCC in people with a positive family history of melanoma. However, a history of previous melanoma is associated with an increased risk of developing SCC and BCC,^15^ and individuals with prior SCC or BCC are at higher risk of developing melanoma.^16^

Few studies have evaluated heterogeneity in risk among melanoma, SCC, and BCC cases from a single population.^17^ In this study, we prospectively examined the associations between phenotypic host factors and the risk of incident melanoma, SCC, and BCC in a cohort of women. The goal was to distinguish the impact of phenotype on the risk of each of these 3 common skin cancers and to replicate previous findings in a unique US population with high follow-up rates.

## METHODS

### Study cohort

We used data from a prospective cohort study, the Nurses’ Health Study (NHS),^3^ which was established in 1976 with 121 700 female nurses aged 30 to 55 years in 11 US states. We have ascertained their lifestyle habits and disease history every 2 years since 1976 via mail questionnaires. Skin cancer has been assessed every 2 years since 1984, and skin cancer risk factors were assessed in 1982, 1986, and 1992 (see “Exposure assessment” below). Details of this cohort have been described previously.^18^^,^^19^

### Study population

We conducted a prospective study from 1984–2002. A total of 113 139 participants were followed over the 18-year period. Women with a history of any cancer before 1984 were excluded, as were women who had more than 1 type of skin cancer at any point during the 18-year follow-up, as determined by self-report or case confirmation by medical record review (eg, a woman with confirmed melanoma and SCC would have been excluded). Almost all subjects were US non-Hispanic whites. Appropriate research approval for institutional human studies was obtained at the Brigham and Women’s Hospital.

### Case ascertainment

Skin cancer confirmation is routinely conducted. For all 3 skin cancers, participants report new cases during each 2-year cycle. For reported SCC and melanoma cases, we reviewed ICD-9 codes in medical records to confirm the diagnosis. SCC in situ, actinic keratoses, SCC of the oral mucosa or genitalia, melanoma in situ, and dysplastic nevi were excluded from this analysis. Medical records are not obtained for self-report of BCC. Colditz et al^20^ performed a validation study in 1986 and demonstrated that the validity of self-reports of BCC was higher than 90%, as confirmed by histopathologic records, and this high validity of self-reports of BCC was independently confirmed in a subsequent study.^21^

### Exposure assessment

All information on risk factors and exposures was collected via biennial questionnaires. In 1982, the following information was collected: (1) natural hair color at age 20 years (hair color), (2) as a child or adolescent, the reaction the skin had after 2 or more hours in the sun on a bright sunny day after exposure to the sun several times (susceptibility to burn), (3) as a child or adolescent, the kind of tan that developed after repeated sun exposures, eg, a 2-week vacation outdoors (ability to tan), and (4) family history of melanoma in first-degree relatives.^22^ In 1986, data were collected on the number of nevi measuring 3 mm or larger on the left arm from the shoulder to the wrist. In 1992, data on family history was collected again.

### Statistical analysis

For the age-adjusted (5-year categories) and multivariate regression models, variables were modeled as dichotomous^22^ or categorical. We selected the categories based on the original questions asked in 1982 and 1986. Each study participant contributed person-time from the date the 1984 questionnaire was returned to the date of an incident melanoma, SCC, or BCC, or 30 June 2002, whichever came first. The category with the lowest risk of skin cancer was always used as the reference group. Hence, “black hair” was the reference group for the variable “natural hair color at age 20” and “no burn” was the reference category for the variable “susceptibility to burn” (Table 2). We calculated relative risks by using Cox proportional hazards regression to adjust for age and other potential confounders, including family history of melanoma, hair color at age 20 years, number of moles on left upper arm, ability to tan, and susceptibility to burn. To test for trend, we modeled the exposures as ordinal variables.

**Table 1.** Baseline characteristics of study participants (1984–2002)

	Person-years	Melanoma		Squamous cell carcinoma		Basal cell carcinoma	
			
		Cases	Crude incidence rate^a^	Cases	Crude incidence rate^a^	Cases	Crude incidence rate^b^
Total	1 814 108	375	20.67	495	27.29	9423	519.43
Age (years, mean)^b^		58.54		61.40		63.73	
Family history of melanoma							
No	1 725 247	341	19.77	473	27.42	8746	506.94
Yes	88 861	34	38.26	22	24.76	677	761.86
Number of moles on left arm							
0	793 854	138	17.38	251	31.62	4288	540.15
1–2	396 930	114	28.72	136	34.26	2571	647.72
3–5	31 859	19	59.64	14	43.94	243	762.74
≥6	23 958	15	62.61	11	45.91	180	751.31
Childhood/adolescent tanning ability							
Good tan	334 653	49	14.64	79	23.61	1462	436.87
Average tan	631 873	136	21.52	175	27.70	3503	554.38
Little tan	305 274	93	30.46	121	39.64	2054	672.84
Almost none	119 761	37	30.89	53	44.25	849	708.91
Childhood/adolescent burn susceptibility							
No burn	288 546	46	15.94	60	20.79	1072	371.52
Some redness	613 152	124	20.22	170	27.73	3247	529.56
Burn	306 377	90	29.38	129	42.10	2117	690.98
Painful burn	130 844	36	27.51	50	38.21	953	728.35
Painful burn with blisters	69 697	26	37.30	26	37.30	588	843.65
Natural hair color at age 20 years							
Black	60 318	8	13.26	13	21.55	193	319.97
Dark brown	601 502	112	18.62	169	28.10	2966	493.10
Light brown	532 894	139	26.08	157	29.46	3170	594.87
Blonde	157 085	43	27.37	68	43.29	1172	746.09
Red	56 347	19	33.72	26	46.14	476	844.77

^a^mean age at time of diagnosis.

^b^per 100 000 person-years.

To test for differences in associations with melanoma, SCC, and BCC, we used polytomous logistic regression.^23^ We used a custom software program described by Marshall and Chisholm,^24^ which permits formal testing of the differences in estimating the beta of each risk factor for the separate components of a composite endpoint. It can specify the variables modeled with a common beta estimate for all outcomes and those modeled with distinct values. We modeled 4 outcome categories (non-diseased, melanoma cases, SCC cases, and BCC cases) using polytomous regression. All risk estimates for each exposure variable were allowed to vary between the 4 outcomes in the initial model. Then, a manual stepwise procedure was conducted, each time with 1 risk factor constrained to be uniform across the 4 endpoints while all others were allowed to vary. The likelihood ratio test (LRT) was used to evaluate the appropriateness of modeling each exposure variable as the same for all 4 outcomes. In comparing each successive model with the baseline model, the variable with the highest LRT *P*-value was set to have 1 common estimate for melanoma, SCC, and BCC, and we used this as the baseline model for the next set of models. For each of the remaining variables, we repeated this procedure and set more variables to be the same for the 4 outcomes. This was continued until all remaining variables had LRT *P*-values less than or equal to 0.05, which indicated that they were likely to have different associations with the 3 outcomes. We simplified the expressions of each variable for the polytomous logistic regression to reduce the complexity of the computations. Age was modeled as a continuous variable (with 1-year increments) and family history as a dichotomous variable (unchanged from the logistic regression models). For number of nevi, susceptibility to burn, ability to tan, and hair color, we used ordinal variables. Because the risk estimates from polytomous logistic models and logistic models cannot be directly compared, the results from the 2 models are presented separately in Tables 2 and 3.

**Table 2.** Age-adjusted and multivariate analyses of risk factors for melanoma, squamous cell carcinoma, and basal cell carcinoma

	Melanoma			Squamous cell carcinoma			Basal cell carcinoma		
			
	*n*^a^	RR (95% CI)	RR (95% CI)	*n*	RR (95% CI)	RR (95% CI)	*n*	RR (95% CI)	RR (95% CI)
		Age-adjusted^b^	Multivariate^c^		Age-adjusted	Multivariate		Age-adjusted	Multivariate
Family history^d^	34	1.94 (1.36, 2.76)	1.71 (1.20, 2.43)	22	0.82 (0.58, 1.37)	0.79 (0.52, 1.22)	677	1.49 (1.38, 1.62)	1.37 (1.26, 1.48)
*P* value		0.0002	0.003		0.6	0.29		<0.0001	<0.0001
Ability to tan^e^									
Referent (‘good tan’)	49	1.00	1.00	79	1.00	1.00	1462	1.00	1.00
Average tan	136	1.46 (1.05, 2.03)	1.28 (0.91, 1.79)	175	1.15 (0.88, 1.50)	1.02 (0.77, 1.34)	3503	1.26 (1.18, 1.33)	1.05 (0.95, 1.16)
Little tan	93	2.09 (1.48, 2.95)	1.61 (1.10, 2.37)	121	1.68 (1.27, 2.23)	1.32 (0.96, 1.82)	2054	1.57 (1.47, 1.68)	1.13 (1.05, 1.22)
Almost none	37	2.08 (1.36, 3.19)	1.55 (0.95, 2.53)	53	1.80 (1.27, 2.55)	1.41 (0.94, 2.11)	849	1.60 (1.47, 1.74)	1.07 (1.00, 1.14)
*P* for trend		0.8	0.02		0.15	0.04		0.003	0.12
Moles on left arm^f^									
Referent (none)	138	1.00	1.00	251	1.00	1.00	4288	1.00	1.00
1–2 moles	114	1.67 (1.30, 2.14)	1.62 (1.26, 2.07)	136	1.12 (0.90, 1.37)	1.10 (0.89, 1.35)	2571	1.23 (1.17, 1.29)	1.20 (1.14, 1.26)
3–5 moles	19	3.47 (2.15, 5.60)	3.29 (2.04, 5.33)	14	1.45 (0.85, 2.48)	1.40 (0.82, 2.41)	243	1.49 (1.31, 1.70)	1.43 (1.26, 1.63)
≥6 moles	15	3.66 (2.15, 6.24)	3.48 (2.04, 5.94)	11	1.53 (0.83, 2.79)	1.48 (0.81, 2.71)	180	1.48 (1.28, 1.72)	1.42 (1.22, 1.64)
*P* for trend		<0.0001	<0.0001		<0.0001	<0.0001		<0.0001	<0.0001
Susceptibility to burn^g^									
Referent (no burn)	46	1.00	1.00	60	1.00	1.00	1072	1.00	1.00
Some redness	124	1.26 (0.90, 1.77)	1.10 (0.78, 1.55)	170	1.32 (0.98, 1.76)	1.26 (0.93, 1.70)	3247	1.42 (1.32, 1.52)	1.33 (1.24, 1.42)
Burn	90	1.83 (1.29, 2.62)	1.35 (0.92, 1.99)	129	1.99 (1.47, 2.71)	1.69 (1.21, 2.35)	2117	1.87 (1.74, 2.01)	1.64 (1.52, 1.78)
Painful burn	36	1.73 (1.12, 2.68)	1.19 (0.74, 1.91)	50	1.86 (1.27, 2.70)	1.45 (0.96, 2.18)	953	2.02 (1.85, 2.21)	1.73 (1.58, 1.91)
Painful burn with ​ blisters	26	2.31 (1.43, 3.74)	1.52 (0.89, 2.60)	26	1.75 (1.10, 2.77)	1.29 (0.78, 2.15)	588	2.31 (2.08, 2.55)	1.95 (1.74, 2.18)
*P* for trend		<0.0001	0.07		<0.0001	0.04		<0.0001	<0.0001
Hair color^h^									
Referent (black)	8	1.00	1.00	13	1.00	1.00	193	1.00	1.00
Dark brown	112	1.45 (0.71, 2.96)	1.33 (0.64, 2.72)	169	1.40 (0.80, 2.46)	1.30 (0.74, 2.30)	2966	1.64 (1.42, 1.90)	1.50 (1.30, 1.74)
Light brown	139	2.01 (0.99, 4.11)	1.68 (0.82, 3.44)	157	1.44 (0.82, 2.54)	1.24 (0.70, 2.20)	3170	1.96 (1.70, 2.27)	1.68 (1.45, 1.95)
Blonde	43	2.09 (0.98, 4.45)	1.62 (0.75, 3.47)	68	2.06 (1.14, 3.72)	1.65 (0.90, 3.01)	1172	2.42 (2.08, 2.82)	1.96 (1.68, 2.29)
Red	19	2.58 (1.13, 5.89)	1.74 (0.75, 4.06)	26	2.21 (1.14, 4.31)	1.53 (0.77, 3.03)	476	2.78 (2.35, 3.28)	2.00 (1.69, 2.38)
*P* for trend		0.17	0.06		0.01	0.08		0.001	<0.0001

Abbreviations: CI, confidence interval; RR, relative risk.

^a^Number of participants does not sum to total due to missing data.

^b^Age-adjusted in 5-year categories.

^c^Multivariate models include the following covariates: age, family history of melanoma, hair color at age 20 years, number of moles on left upper extremity, ability to tan, and susceptibility to burn.

^d^Family history of melanoma (first-degree relative), dichotomous variable with yes/no response.

^e^As a child or adolescent, what kind of tan developed after repeated sun exposures, eg, a 2-week outdoor vacation.

^f^Number of moles (size ≥3 mm) on the left arm from the shoulder to the wrist.

^g^As a child or adolescent, what kind of reaction would the skin have after 2 or more hours in the sun on a bright sunny day after exposure to the sun several times.

^h^Natural hair color at age 21.

## RESULTS

During the 18-year follow-up period, there were 375 cases of melanoma, 495 cases of SCC, and 9423 cases of BCC (Table 1). All cases were incident primary skin cancers diagnosed after 1984. Mean age at diagnosis was lowest for the melanoma cases (58.54 years), followed by the SCC (61.40 years) and BCC (63.73 years) cases. The proportion of women with a positive family history of melanoma was highest among those who developed melanoma (9%), followed by BCC (7%) and SCC (4%). For nevus counts on the left arm, 4% of women with melanoma had 6 or more nevi, as compared with 2% each for SCC and BCC cases. Among the melanoma, SCC, and BCC cases, there was no substantial difference in any other risk factor at baseline, including susceptibility to burn and natural hair color at age 20.

**Table 3.** Multivariate^a^ relative risks for melanoma, squamous cell carcinoma (SCC), and basal cell carcinoma (BCC) in multinomial regression models

	Melanoma	Squamous cell carcinoma	Basal cell carcinoma	*P* value^b^
			
	RR (95% CI)	RR (95% CI)	RR (95% CI)	
Age^c^	1.04 (1.03, 1.06)	1.01 (1.00, 1.02)	1.05 (1.04, 1.05)	<0.0001
Moles on left arm	1.36 (1.22, 1.52)	1.29 (1.17, 1.42)	1.17 (1.15, 1.20)	0.007
Family history of melanoma	1.72 (1.21, 2.44)	1.23 (0.80, 1.89)	1.37 (1.27, 1.49)	0.016
Tan after sun exposure^d^		1.00 (0.98, 1.03)		0.097
Burn after sun exposure^d^		1.15 (1.12, 1.17)		0.715
Hair color^d^		1.07 (1.05, 1.09)		0.641

Abbreviations: CI, confidence interval; RR, relative risk.

^a^Multivariate polytomous regression; age was included as a continuous variable in 1-year increments (*P* < 0.0001); family history remained a dichotomous variable, the remaining variables were ordinal.

^b^The control group for polytomous (multinomial) logistic regression analysis was women with no skin cancer.

^c^1-year increments in age.

^d^Single effect estimates and 95% confidence intervals are presented because there was no significant difference among melanoma, SCC, and BCC for these risk factors.

Table 2 shows the results of age-adjusted and multivariate analyses of skin cancer phenotypic factors, ie, family history of melanoma, ability to tan after 2 hours of sun exposure, number of nevi on the left arm, susceptibility to burn after 2 hours of sun exposure, and natural hair color at age 20. In age-adjusted analyses, differences were noted between melanoma and SCC for family history of melanoma (RR 1.94, 95% CI 1.36–2.76, and RR 0.82, 95% CI 0.58–1.37, respectively) and 6 or more nevi on the left arm (RR 3.66, 95% CI 2.15–6.24) and RR 1.53, 95% CI 0.83–2.79, respectively). In multivariate analyses, family history of melanoma and number of nevi on the left arm remained significant for melanoma and nonsignificant for SCC. Regarding susceptibility to burn, a painful burn with blisters was associated with a higher risk of melanoma (RR 2.31, 95% CI 1.43–3.74) as compared with SCC (RR 1.75, 95% CI 1.10–2.77), but this association was nonsignificant in multivariate analyses. The red-hair phenotype was not associated with a higher age-adjusted risk of melanoma (RR 2.58, 95% CI 1.13–5.89) as compared with SCC (RR 2.21, 95% CI 1.14–4.31) and was nonsignificant in multivariate analyses. Blonde hair color did not materially influence the risk of SCC versus that of melanoma.

The findings for melanoma and BCC were similar with respect to hair color, susceptibility to burn, and family history of melanoma. Age-adjusted and multivariate estimates for number of nevi on the left arm, susceptibility to burn, and lighter hair color were significant for BCC. As compared with women with SCC, those with melanoma and BCC (RR 2.31, 95% CI 2.08–2.55) were equally likely to report painful burns with blisters after sun exposure. Interestingly, women with BCC (RR 2.78, 95% CI 2.35–3.28), melanoma, and SCC were equally likely to have red hair. However, for family history of melanoma, the risk estimates were lower for BCC in both age-adjusted (RR 1.49, 95% CI 1.38–1.62) and multivariate analyses (RR 1.37, 95% CI 1.26–1.48). Unlike melanoma, age-adjusted estimates for “almost no” ability to tan were lower (RR 1.60, 95% CI 1.47–1.74) and remained marginally significant in multivariate models (RR 1.07, 95% CI 1.00–1.14).

Heterogeneity between melanoma, SCC, and BCC was evaluated using polytomous regression (PLR) models, and the results are presented in Table 3. Using PLR, the risk of each type of skin cancer was compared with a common non-cancer control group, and the relative differences between cancers were evaluated. Significant *P*-values for family history of melanoma (*P* = 0.016) and nevi on the left arm (*P* = 0.007) indicate that there were statistically significant differences among melanoma, SCC, and BCC cases in these 2 phenotypic characteristics. Although risk estimates obtained from PLR models cannot be compared directly with the findings in Table 2, estimates for both family history and number of nevi were higher for melanoma as compared with SCC and BCC. There was no significant difference among the 3 skin cancer types in susceptibility to burn or hair color, whereas there was a tendency towards a difference in ability to tan (*P* = 0.097).

## DISCUSSION

In this prospective study of US women, we have shown that host factors are not associated with identical risks of incident melanoma, SCC, and BCC. By identifying women with only 1 type of incident skin cancer^25^ and comparing their risk with that of other women with only first SCC or first BCC in a single cohort, we removed the variability in populations and data quality that complicates comparisons of separate case-control studies of the 3 cancer types. To evaluate phenotypic differences among women who developed melanoma versus those who developed SCC or BCC, we excluded women who developed more than 1 type of skin cancer and studied women who had only 1 type of skin cancer. It is likely that women with more than 1 type of skin cancer were susceptible to all forms of skin cancer or had heavier sun exposure. The principal goal of this study was to evaluate differences in phenotypic risk between the 3 skin cancers rather than susceptibility to all 3 cancers or environmental exposure. We were then able to use PLR to test for differences in the impact of the different phenotypes on the risk of melanoma, SCC, and BCC.

Family history of melanoma and mole counts on the left arm were associated with an increased risk of melanoma in this study, as was expected, and a lower but still statistically significant risk of BCC. However, family history of melanoma did not affect the risk of SCC, and PLR analysis showed that this risk factor had a significantly different impact on the risk of the 3 skin cancer types. The risk of a high mole count significantly differed among the 3 groups, which suggests that there may be common pathways that are associated with melanoma and BCC development. Interestingly, melanoma, BCC, and nevi develop in the basal layer of the epidermis, although melanoma and nevi arise from melanocytes and BCC arises from keratinocytes. We postulate that a similar microenvironment in the basal layers of the epidermis promotes development of these 3 skin lesions: a benign UV-induced nevus, a malignant melanocyte tumor, and a malignant keratinocyte tumor. It is plausible that UV radiation reaching these deeper layers is of a longer wavelength (more UV-A than UV-B),^26^^,^^27^ that similar types and concentrations of free radicals are generated in these layers,^27^ and that constitutive pigmentation affects the amount of radiation reaching the deeper layers.^28^^,^^29^ Further study of these mechanisms may increase our understanding of the risk of melanoma and BCC and help us to identify individuals before they develop potentially life-threatening skin cancer.

Ability to tan was not a differentiating risk factor for melanoma, SCC, or BCC. PLR analysis showed a trend toward significance in the comparison of the 3 cancer types, although it did not demonstrate a significant difference between the 3 skin cancers in susceptibility to burn or hair color. Interestingly, the red-hair phenotype did not have a differentially higher effect on the risk of melanoma versus that of SCC or BCC. Family history of melanoma and nevus counts emerged as significant risk factors for melanoma as compared with SCC and BCC in this analysis.

Our study was restricted to an occupational cohort of women. Hence, we cannot generalize these results to men. Eye (iris) color was not included as a risk factor in the analysis, as these data were not available. A major strength of the study was collection of phenotype information in 1982 (hair color, ability to tan, susceptibility to burn, family history of melanoma) before development of skin cancer (follow-up began in 1984); hence, the results are unlikely to be affected by recall bias, which can occur in retrospective studies. Another strength of the study is the occupational homogeneity of the cohort and, hence, the limited variability in occupational sun exposure. In addition, the study was sufficiently powered to detect small differences in risk among the factors studied, as indicated by the narrow confidence intervals. Although the question regarding number of nevi was asked in 1986, assessment was retrospective for only 2 of the 18 years of follow-up. All cases of melanoma and SCC were confirmed via review of medical records, thereby reducing misclassification of the outcome. Although BCC was self-reported, available evidence indicates that the validity of self-reports of BCC is higher than 90% in this medically sophisticated cohort.^21^

These findings suggest that there are modest differences in risk between the 3 types of skin cancer. The risks associated with hair color and susceptibility to burn were similar across the 3 cancers. Risk prediction algorithms and clinicians should weigh the risk factors differentially, particularly family history of melanoma and nevus counts. A better understanding of the different risk profiles of these cancers will permit more accurate identification of susceptible populations and help target prevention strategies.
